# Cost-effectiveness of tax policies on promoting sustainable diets in Iran: a modeling study

**DOI:** 10.3389/fnut.2024.1453969

**Published:** 2024-10-21

**Authors:** Amin Mokari-Yamchi, Nasrin Omidvar, Manoochehr Karami, Morteza Tahamipour Zarandi, Hassan Eini-Zinab

**Affiliations:** ^1^Maternal and Childhood Obesity Research Center, Urmia University of Medical Sciences, Urmia, Iran; ^2^Department of Community Nutrition, National Nutrition and Food Technology Research Institute, Faculty of Nutrition Sciences and Food Technology, Shahid Beheshti University of Medical Sciences, Tehran, Iran; ^3^Department of Epidemiology, School of Public Health and Safety, Shahid Beheshti University of Medical Sciences, Tehran, Iran; ^4^Department of Economics, Faculty of Economics and Political Science, Shahid Beheshti University, Tehran, Iran

**Keywords:** tax policy, sustainable diets, cost-effectiveness, Iran, modeling study

## Abstract

**Background:**

Implementation of food taxes may promote sustainable diets in a society. This study estimates the potential short-term impacts of taxes on sugar and sweets (SAS), sugar sweetened beverages (SSB) and hydrogenated oil and animal fats (HOAF) in Iran through a social cost-effectiveness analysis.

**Methods:**

In this study, three tax scenarios were evaluated, including a 25% tax on SASs, a 30% tax on SSBs, and a 30% tax on HOAFs. The data from Iran’s 2019–2020 Household Income and Expenditure Survey (HIES) were utilized, and a simulated population of 1 million individuals aged over 25 years was analyzed. Population impact fraction (PIF) was calculated to estimate the averted number of cases and Disability-Adjusted Life Years (DALYs) under each policy scenario. Additionally, the study assessed water and carbon footprints, as well as all associated costs. Cost-effectiveness was evaluated through incremental cost-effectiveness ratios (ICER) and comparison with WHO-recommended thresholds.

**Results:**

Implementation of taxes on SASs and HOAFs resulted in reductions of 1.09 and 1.08% in water footprint, as well as 0.47 and 1.05% in carbon footprint, respectively. In terms of population health, the interventions resulted in averting 343.92 DALYs (95% UI = 318.62–369.36) for the SSB tax and 1219.01 DALYs (95% UI = 1123.05–1315.77) for the tax on HOAFs. Additionally, the tax on SASs averted 1028.09 DALYs (95% UI = 947.16–1,109). All scenarios were deemed cost-effective based on the WHO threshold for ICER, with values of 0.26 billion Rials/DALY, 0.54 billion Rials/DALY, and 0.17 billion Rials/DALY, respectively.

**Conclusion:**

The studied tax scenarios could generate substantial health gains and be cost-effective in Iran. It is recommended that policymakers consider implementing such price policies to promote healthy and sustainable diets.

## Background

Dietary patterns are changing worldwide, shifting from a plant-based diet with fresh, unprocessed foods to diets with high sugar, fat, and animal products, including ultra-processed food items (1). Consumption of unhealthy foods and beverages is one of the main risk factors of Non-communicable diseases (NCDs), making up nearly 10% of global disease burden (2). The production, processing, distribution, and consumption of food have significant implications for both human health and the environment. Food consumption is responsible for over one-third of global greenhouse gas (GHG) emissions (3). Recognizing the impact of diet on both human health and the planet has highlighted the importance of adopting sustainable dietary practices. As defined by the Food and Agriculture Organization of the United Nations (FAO), Sustainable diets are those that are nutritionally adequate, culturally acceptable, economically accessible, and environmentally sustainable (4). In response to the growing evidence of a causal relationship between unsustainable diets and increased risk of NCDs and environmental crises, governments are increasingly interested in implementing fiscal policies (taxes and subsidies) to promote sustainable food consumption (5).

Governments may adopt policy measures to encourage healthy and sustainable eating choices. Evidence from systematic reviews suggests that providing subsidies for healthy foods and imposing taxes on unhealthy foods can be effective in encouraging better dietary habits and improving health (6, 7). Modeling studies suggest that applied taxes by governments based on greenhouse gas (GHG) emissions can effectively shift dietary behaviors toward foods with a lower environmental impact (8, 9). The study conducted by Springmann et al. revealed that implementing global taxes on GHG emissions related to diet could potentially lead to a 9.6% reduction in emissions from food production, while also preventing approximately 500,000 deaths annually (10). According to the research findings, changes in prices caused by taxation or subsidies can modify people’s consumption habits and potentially result in improved diet and health outcomes (11, 12). Numerous recent studies have analyzed consumer price elasticity to assess how price fluctuations affect the demand for specific food categories, like sugar-sweetened beverages (SSBs) and sugar (13, 14). A cost-effectiveness model study conducted by Cobiac et al., based on price elasticity data demonstrated that tax interventions on saturated fat, SSB, salt and sugar on Australian population were cost-saving from health sector perspective (15), additionally, a tax of $0.01 per ounce on SSBs was shown to be potentially cost-saving and leading to significant improvements in population health and savings in disease treatment costs in the United States (US) (16). Broeks et al. conducted a cost–benefit analysis from a societal perspective, examining the broader impacts of implementing a tax on meat and a subsidy on fruits and vegetables in the Netherlands. The results indicated net welfare gains for Dutch society (17).

Another challenge in determining the cost-effectiveness of food price policies is the potential substitution effect. For instance, if the price of SSBs is increased, there is a possibility that individuals might opt for purchasing more processed foods instead, resulting in no significant improvement in health or environmental effects (18). In order to address this issue, our previously published study employed the Almost Ideal Demand System (AIDS) estimation to assess the impact of various fiscal policies on total purchases patterns and food basket of households (19). By analyzing changes in food groups, total nutrients, and water and carbon footprints under different policies, we aimed to understand how these policies can influence health and the environment. To achieve this, we conducted current modeling study to evaluate the short-term impacts of different tax scenarios on the reduction of incidence rates for diet-related diseases, environmental costs, and the number of averted disability-adjusted life years (DALYs). Furthermore, we analyzed the cost-effectiveness of implementing these scenarios in Iran.

## Materials and methods

### Study overview

In Iran, fiscal policies have not been employed to address nutritional risk factors and environmental considerations for a significant period of time. This is despite the fact that a “nutrition transition” became more pronounced in the early 1980s. During this period, certain energy-providing foods received subsidies, leading to increased consumption of fats and carbohydrates in households’ food baskets (20). Within this context, expert consensus has identified three tax scenarios (19): (1) a 25% tax on sugar and sweets (SASs), (2) a 30% tax on sugar-sweetened beverages (SSBs), and (3) a 30% tax on hydrogenated oils and animal fats (HOAFs). Data from Iran’s 2019–2020 Household Income and Expenditure Survey (HIES), conducted annually by the Statistical Center of Iran, were utilized. The HIES provides information on income and expenditure patterns. Price elasticity data from our previous study were employed to assess the impact of each tax scenario on daily food intake (19). Accounting for food waste, the actual amount of food consumed was estimated based on the FAO recommended waste percentage for each food group during consumption (21). Dietary analyses were conducted using Nutritionist IV software, with energy and nutrient intakes calculated manually. Estimates were determined per Adult Male Equivalent (AME) unit, which represents the energy needs of a household member based on their age and gender compared to an adult male aged 18–30 years with moderate physical activity (22).

### Change in dietary risk factor exposure and disease incidence

**Figure 1:**
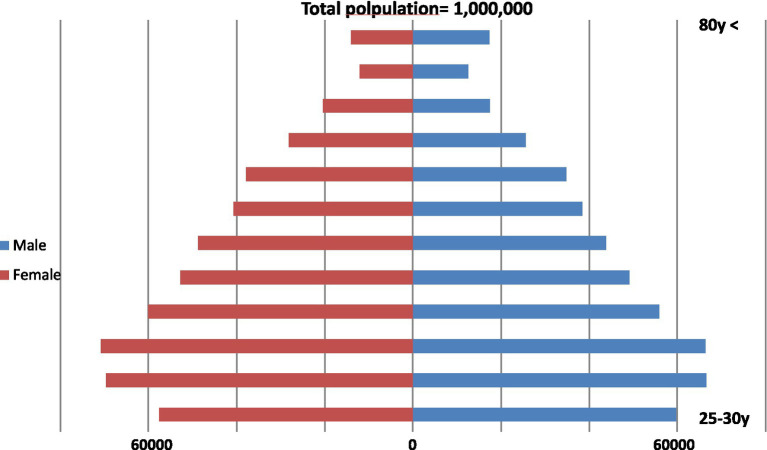
Age and sex structure of the hypothetical population of 1 million Iranians over 25 years old in 2019–2020.

In this research, we used a simulated population of 1 million individuals aged 25 years and above, with a similar age-sex distribution as the Iranian population in 2020 (Figure 1). The aim was to analyze various scenarios by examining changes in food choices and their impact on nutritional risk factors, including SSBs, sugar, saturated and trans fatty acids, as well as BMI. Changes in BMI were calculated based on the modeled alterations in energy intake, based on the methodology outlined in a previous study by Hall et al. (23), while assuming consistent height and physical activity levels. This method was applied to the Iran 2016 STEPS Survey data (24) for individuals of the same age and sex.

The effects of alterations in dietary risk factors on six diet-related diseases, including ischemic heart disease, ischemic stroke, osteoarthritis, type 2 diabetes, colorectal cancer, and stomach cancer were calculated (25–28). To this aim the incidence of these diseases and their corresponding DALY rates were determined by considering sex and age groups, utilizing the 2019 Global Burden of Disease (GBD) health records for the Iranian population (29). In order to evaluate the changes in disease incidence and DALYs for each scenario, the population impact fraction (PIF) was used, which defined as the proportional change in disease risk due to change in exposure to a related risk factor (30). To obtain proportions and number of cases prevented under each intervention scenario, the PIF was calculated for each age and sex using the following equation (31):


$$RR\left(M_{i}\right)=\exp\left(\frac{Ln\left(RR_{\mathrm{Per}\;x\;\mathrm{gram}}\right)}{x}.M_{i}\right)$$

$$PIF=1-\overset{n}{\underset{i}{\sum }}RR\left(M_{i}^{*}\right)/\overset{n}{\underset{i}{\sum }}RR\left(M_{i}\right)$$

Where $M_{i}$ is the mean exposure level (in grams per day) in each individual i, RR is the corresponding relative risk for that individual, and $M_{i}^{*}$ is the mean exposure level of individual i after the change in risk factor due to a specific intervention. The estimated number of averted DALYs under each policy scenario was calculated by applying the PIF to the corresponding predicted number of DALYs for each age and sex group. The data were analyzed in the Microsoft Excel version 2016.

### Environmental footprint

In this study, the environmental aspect of sustainable diets was evaluated using water and carbon footprints. The water footprint represents the amount of freshwater utilized in the production of goods and services consumed by individuals or communities. Data on the water footprint of Iran were obtained for analysis (32, 33). For assessing the level of emissions, the carbon footprint method was applied, which measures the total amount of carbon dioxide emitted directly or indirectly as a result of an activity or throughout the entire life cycle of a product (34). The required data on the carbon dioxide emissions of each food items were also obtained from the BCFN double pyramid database (35).

### Calculating costs and cost-effectiveness

In this study, the cost of implementing the proposed taxes were estimated from a societal perspective and three cost section were included: 1. Household Costs: These reflect the financial burden on households, calculated based on the price difference between the basic food basket and the modified food basket after applying the tax scenarios. It is important to note that the tax itself was not included in this calculation. Instead, we focused on how the tax influences market prices, the total changes in the items purchased within the household food basket, and, consequently, household expenditures, 2. Health-related costs included direct medical costs (e.g., cost per averted case of stroke), were calculated by using previous data on disease costs in Iran (36–41), 3. Environmental costs, determined by considering the social cost of carbon and agricultural water prices associated with the food items in households’ food baskets (42) were calculated. All costs were inflated to 2020 Iran Rials using the Consumer Price Index (CPI) (43).

To evaluate the cost-effectiveness of the different intervention scenarios, the incremental cost-effectiveness ratios (ICERs) were calculated. The ICERs were obtained by taking the change in total costs and dividing it by the change in DALYs averted for a hypothetical population of 1 million Iranians following the implementation of tax scenarios. The ICER provides cost per DALY averted compared to the other alternatives or with a cost-effectiveness threshold (44). To determine cost-effectiveness, we compared the ICERs with the WHO-recommended threshold Human Development Index (HDI) for low and medium countries (45). If the ICER was lower than the GDP *per capita* of Iran in 2020 (0.61 billion Rials) (46), the intervention scenario was considered cost-effective.

### Sensitivity and uncertainty analyses

Probabilistic sensitivity analyses were conducted by four scenarios of interest. One-way sensitivity analyses were evaluated to test the cost-effectiveness of lower (10%) and higher (50%) food tax levels for the interventions. Furthermore, alternative assumptions regarding environmental costs were explored by incorporating accounting approach (47) and industrial prices (48) of water instead of agricultural prices in Iran. The ICERs were calculated by considering variations in tax rates and revised net costs.

## Results

Table 1 displays the average percentage changes in total nutritional risk factors resulting from tax policy interventions. For example, the SASs tax produced a 10.76% (95% uncertainty interval [UI] 10.48 to 11.03) decrease in sugar, and the SSB fat tax gave 14.26% (13.87–14.67) reduction in SSB and the HOAF tax resulted in a 7.82% (7.45–8.18) and 15.84% (13.94–17.73) reduction in saturated and trans fatty acids, respectively. The midpoint BMI decreased across all scenarios, with taxation on SASs, SSBs, and HOAFs resulting in mean BMI reductions of 0.45% (0.39–0.48), 0.12% (0.11–0.13), and 0.44% (0.39–0.49), respectively in the population (Table 1).

**Table 1:** Average percentage changes in total nutritional risk factors following tax policy interventions.

Tax policy option	Sugar	SSB	SFA	TFA	BMI
Sugar and sweets tax	−10.76% (−11.03 to −10.48)	1.24% (1.12–1.35)	−0.4% (−0.43 to −0.36)	−0.6% (−0.66 to −0.53)	−0.45% (−0.48 to −0.39)
Sugar sweetened beverages tax	−1.96% (−2 to −1.91)	−14.26% (−14.67 to −13.87)	0.63% (0.59–0.67)	0.78% (0.7–0.86)	−0.12% (−0.13 to −0.11)
Hydrogenated oil and animal fats tax	−1.97% (−2.1 to −1.92)	−2.11% (−2.21 to −2)	−7.82% (−8.18 to −7.45)	−15.84% (−17.73 to −13.94)	−0.44% (−0.49 to −0.39)

Values are presented as mean (95% uncertainty interval). SSB, sugar sweetened beverage; SFA, saturated fatty acids; TFA, trans fatty acids; BMI, body mass index.

We calculated the percentage differences in response to the scenarios for selected age-standardized disease incidence rates (Table 2). Diabetes incidence decreased more than any other disease for all tax interventions, ranging from a 1.22% fall for the SSB tax up to a 2·52% fall for the SASs tax.

**Table 2:** Percentage changes in age and sex-standardized incidence rates for selected diseases following tax policy interventions.

Tax policy option	Ischemic heart disease	Diabetes type 2	Ischemic stroke	Stomach cancer	Colorectal cancer	Osteoarthritis
Sugar and sweets tax	−0.97% (−1.08 to −0.85)	−2.52% (−2.8 to −2.24)	−0.01% (−0.012 to −0.007)	−0.4% (−0.44 to −0.36)	−0.43% (−1.03 to −1.48)	−0.93% (−1.01 to −0.85)
Sugar sweetened beverages tax	−0.27% (−0.31 to −0.23)	−1.22% (−1.46 to −0.98)	−0.22% (−0.24 to −0.19)	−0.11% (−0.12 to −0.09)	−0.10% (−0.11 to −0.08)	−0.25% (−0.28 to −0.21)
Hydrogenated oil and animal fats tax	−1.44% (−1.62 to −1.25)	−2.36% (−2.66 to −2.06)	−0.81% (−0.72 to −0.9)	−0.38% (−0.42 to −0.33)	−0.43% (−0.48 to −0.37)	−0.88% (−0.96 to −0.8)

Age-standardizations are to world health organization (WHO) standard.

**Figure 2:**
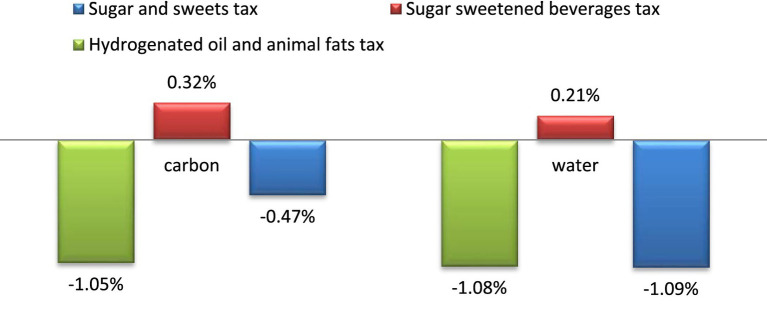
Water and carbon footprint percent changes following tax policy interventions.

Figure 2 presents the average percentage change in water and carbon footprints in different scenarios. The implementation of taxes on SASs and on HOAFs resulted in reductions of 1.09 and 1.08% in water footprint, and 0.47 and 1.05% in carbon footprint, respectively. However, the imposition of tax on SSB had negative environmental consequences, as the water and carbon footprints increased by 0.21 and 0.32%, respectively.

Policy intervention costs for the SAS, SSB and HOAF incentive would be 345.82 and $185.71 and 302.12 billion Rials, respectively. The taxation on SASs resulted in savings of 44.56, 4.36, and 26.17 billion Rials due to reductions in water footprint, carbon footprint, and healthcare costs. Taxation on SSB led to an increase of 8.39 and 2.98 billion Rials in water and carbon footprint costs, while saving 9.46 billion Rials. Similarly, the taxation on HOAF saved 44.46, 9.65, and 31.79 billion Rials due to reductions in water footprint, carbon footprint, and healthcare costs (Table 3).

**Table 3:** Health impacts, costs and cost effectiveness of tax policies interventions through societal perspective in a hypothetical population of 1 million Iranians.

		Sugar tax	Sugar sweetened beverages tax	Hydrogenated oil and animal fats tax
Household costs (BR)		345.82 (332.25, 359.4)	185.71 (176.94, 194.44)	302.12 (291.73, 312.52)
Environmental costs (BR)	Water	−44.56 (−47.12, −41.91)	8.39 (7.94, 8.83)	−44.46 (−46.22, −42.72)
	Carbon	−4.36 (−4.68, −4.06)	2.98 (2.75, 3.2)	−9.65 (−9.1, 10.19)
Healthcare costs (BR)		−26.17 (−29.05, −23.29)	−9.46 (−10.46, −8.44)	−31.79 (−34.61, −28.95)
Total costs (BR)		270.71 (261.6, 279.86)	187.63 (179.93, 195.39)	216.22 (209.78, 222.61)
Total DALYs averted		1028.09 (947.16, 1,109)	343.92 (318.62, 369.36)	1219.01 (1123.05, 1315.77)
ICER, BR/DALY		0.26 (0.23, 0.28)	0.54 (0.51, 0.56)	0.17 (0.15, 0.18)

Values are presented as mean (95% uncertainty interval). All costs are presented in 2020 Iranian Rials. BR, Billion Rials; DALY, disability-adjusted life years; ICER, incremental cost-effectiveness ratio.

For the hypothetical population of 1 million Iranians taxation scenarios led to an improvement in population health, which ranged from 343.92 (95% UI = 318.62–369.36) DALYs averted for the SSB tax up to 1219.01 DALYs averted (95% UI = 1123.05–1315.77) for the HOAFs tax. Additionally, taxing on SASs averted 1028.09 (95% UI = 947.16–1,109) DALYs. From a societal perspective, considering the WHO threshold for ICER, all scenarios involving taxing SASs, SSB, and HOAFs were revealed cost-effective, with ICER values of 0.26 billion Rials/DALY, 0.54 billion Rials/DALY, and 0.17 billion Rials/DALY, respectively (Table 3).

**Figure 3:**
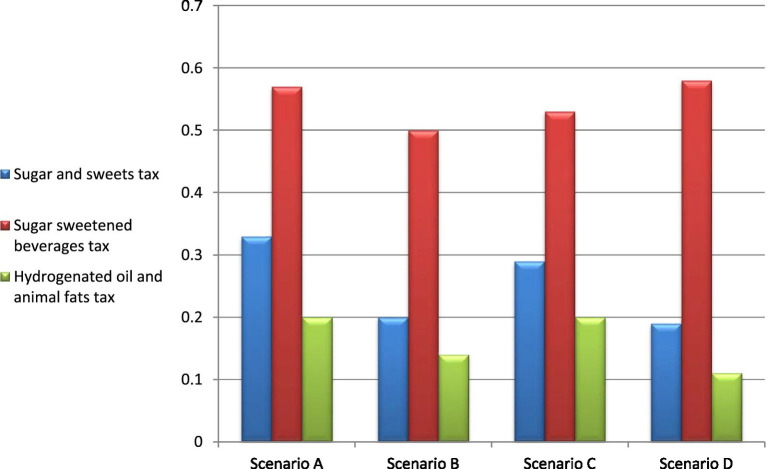
Incremental cost-effectiveness ratio (BR/DALY) analysis in four sensitivity scenarios. Scenario A: 10% tax rate, Scenario B: 50% tax rate, Scenario C: water price based on the accounting value, Scenario D: water price based on industrial value.

In order to evaluate the robustness of our results, we conducted multiple sensitivity analyses. Initially, we computed the ICER for different tax rates (10 and 50%). Subsequently, we examined the impact of varying the cost of water. As illustrated in Figure 3, all scenarios remained cost-effective across different assumptions. When assuming a higher water price for the tax on SASs, as well as HOAFs scenarios, the ICER decreased, while it increased for the SSB tax scenario.

## Discussion

This is the first study in Iran conducted to quantify the effect of price increase in unhealthy food items through employing different tax policies to assess health and environmental benefits, expenditures and cost-effectiveness. The study findings illustrate how tax policy interventions could potentially affect nutritional risk factors, disease incidence rates, environmental impact, healthcare expenses, and overall population health.

The model predicts that in all tax scenarios diabetes incidence fall more than any other disease, with the highest fall for the SASs tax. A similar study conducted by Blakely et al., on the New Zealand population indicated that the majority of the health benefits from tax policies were attributed to changes in BMI, leading to a significant decrease in diabetes incidence (49). The tax on HOAFs produced the highest health gain and health cost savings costs across all scenarios which averted 1,219 DALYs. This outcome may be attributed to the higher own-price elasticity of HOAFs in the Iranian population (19), as well as significant beneficial substitution effects within the food basket in this specific scenario. From the environmental perspective highest water and carbon cost saving was observed in SASs tax and HOAFs tax, respectively. Conversely, the SSB tax had adverse environmental effects. This relates to the substitution effects outlined in our recently published paper, which found that taxation on SSBs resulted in a 13.8% reduction in SSB consumption among households. However, these substitution effects led to increased purchases of red meat, fruit, vegetables, and legumes by 0.46, 1.39, 1.47, and 1.89%, respectively (19). Previous simulation studies have detected similar trends, demonstrating that when food-related fiscal policies are implemented, certain substitutions are anticipated, which can ultimately influence the overall impact of these policies (49, 50).

The findings indicate that implementing taxes on SASs, SSB and HOAFs in Iran can be cost-effective, align with the WHO’s threshold of one GDP *per capita* of Iran. Numerous studies have examined the impacts of targeted food taxes in various settings on consumption, health, and the environment separately. However, fewer studies have considered both health and environmental effects of food taxes in cost-effectiveness analyses. In 2017, Cobiac and his colleagues examined taxes on saturated fat, salt, sugar, and SSBs. They conducted simulations to estimate the total DALYs and costs over the lifespan of the 2010 Australian population from a health perspective. They evaluated the potential cost-effectiveness of each tax option against a threshold of AU$50,000 per DALY averted. Their analysis indicated that all interventions led to cost savings (15). Similar study conducted by Veerman on the impact of a 20% tax on SSBs on the Australian population revealed that modest changes in average body mass resulting from the tax led to significant health benefits. The study estimated gains of 112,000 health-adjusted life years (HALY) for men (95% UI: 73,000–155,000) and 56,000 HALY for women (95% UI: 36,000–76,000). Additionally, the tax was associated with a reduction in overall health care expenditure of AUD609 million (95% UI: 368 million–870 million) (51). Also, Long et al. conducted a study on the impact of a $0.01/ounce tax on SSB in the US population. The result showed a 20% decrease in SSB consumption and a mean BMI reduction of 0.16 kg/m^2^. The implementation of this policy from 2015 to 2025, is projected to prevent 101,000 DALY, generate 871,000 quality-adjusted life years (QALY), and should lead to healthcare cost savings of $23.6 billion (16).

The sensitivity analyses conducted to test the robustness of the findings further support the overall cost-effectiveness of the tax interventions. As demonstrated, higher tax values lead to lower ICER compared to lower tax values. The primary mechanisms driving lower ICER values under higher tax scenarios on SSBs include substantial decreases in consumption, significant health outcome benefits, and lower long-term healthcare costs. In contrast, lower tax levels do not create enough change to achieve similar cost-effectiveness, highlighting the importance of taxation policy as a public health tool. Additionally, the variations in tax rates and water costs did not significantly alter the cost-effectiveness of the scenarios, reinforcing the stability of the results under different assumptions.

## Strengths and limitations

This study has a number of strengths. The consumption data was from a representative sample of the Iranian population. Tax policies were implemented across the entire food basket of households, and the analysis included calculations of water and carbon footprints and nutrients considering all substitution effects. Finally, multiple sensitivity analyses were used to demonstrate the effect of different input parameters and methods on the stability of the results.

There are also several limitations that should be considered in evaluating the results. Firstly, this study focused on the short-term effects of food taxes on health and the environment. It is possible that the observed effects may diminish over time, and then long-term impacts may be better explored in future studies. Secondly, due to data limitation, the relative risk estimates used for calculating the PIF were not specific to Iran, which may introduce some uncertainty in the results. Additionally, the water and carbon footprints of the food items assessed in the study were not specific to Iran, which could potentially impact the accuracy and applicability of the findings in an Iranian context. Thirdly, our cost estimate inputs were based on previous Iranian studies data which is subject to information and selection bias. Finally, we assumed a 100% pass on rate in applying the scenarios which may not reflect the actual implementation and impact in real-world scenarios where pass-on rates could vary.

## Conclusion

In conclusion, our findings suggest that implementing tax policies to encourage healthier eating habits among Iranian adults could generate substantial health and environmental gains and be cost-effective overall. These findings could inform policymakers and stakeholders in developing successful strategies to encourage sustainable diets within the population. Countries with similar contexts could benefit from implementing similar tax policies.

## Data Availability

Publicly available datasets were analyzed in this study. This data can be found at: https://old.sci.org.ir/english.
